# Effect of Traditional Chinese Exercise on Gait and Balance for Stroke: A Systematic Review and Meta-Analysis

**DOI:** 10.1371/journal.pone.0135932

**Published:** 2015-08-20

**Authors:** Bing-Lin Chen, Jia-Bao Guo, Ming-Shuo Liu, Xin Li, Jun Zou, Xi Chen, Ling-Li Zhang, Yu-Shan Yue, Xue-Qiang Wang

**Affiliations:** 1 Department of Sport Rehabilitation, Shanghai University of Sport, Shanghai, China; 2 Second School of Clinical Medical, Nanjing University of Chinese Medicine, Nanjing, China; 3 Bulloch Academy, Statesboro, United States of America; 4 Key Laboratory of Exercise and Health Sciences of Ministry of Education, Shanghai University of Sport, Shanghai, China; 5 Kinesiology and Exercise Science, Shanghai University of Sport, Shanghai, China; 6 Department of Rehabilitation Medicine, Shanghai Shangti Orthopaedic Hospital, Shanghai, China; Cardiff University, UNITED KINGDOM

## Abstract

**Objective:**

A systematic review is conducted to determine the effect of traditional Chinese exercise for patients with stroke.

**Methods:**

Studies are obtained from PubMed, Embase, Cochrane Library, EBSCO, Web of Science, and CNKI. Only randomized controlled trials were left to evaluate the effects of traditional Chinese exercise for patients with stroke, and with no limits on study data or language. The primary outcome was the Berg balance score (BBS), Functional walking scale. And a random-effects model was used to calculate the pooled mean difference (MD) with 95% confidence interval (CI).

**Results:**

A total of 9 studies on 820 participants conform to the inclusion criteria, whereas eight studies on 704 participants are used as data sources for the meta-analysis, all trials were published between 2004 and 2013. The BBS indicates that the efficacy of traditional Chinese exercise on balance of patients with stroke is better than that of other training or no training in short term [MD (95%CI) = 11.85 [5.41, 18.30], P < 0.00001]. And the short physical performance battery, Functional walking scale, limit of stability were observed significant differences on balance (p<0.05) and gait (p<0.05) between traditional Chinese exercise and other exercises or no exercise. In addition, there is an article showed that some other form (physiotherapy exercises focused on balance) significantly improved balance ability for stroke patients compared to tai chi chuan practice (Berg test = 0.01, Romberg, and standing on one leg).

**Conclusion:**

In our meta analysis, the positive findings of this study suggest traditional Chinese exercise has beneficial effects on the balance ability in short term. However, we drew the conclusion according to the extreme heterogeneity, and evidence of better quality and from a larger sample size is required. Because of the inconsistent outcomes, there are short of enough good evidence for patients with stroke to prove the effects of traditional Chinese exercise on gait.

**Systematic Review Registration:**

http://www.crd.york.ac.uk/PROSPERO PROSPERO registration number: CRD42013006474.

## Introduction

Stroke has high morbidity, mortality, and disability rates, and approximately two million people each year suffer from stroke. Consequently, 70%–80% of stroke patients cannot live independently because of disability [1]. In 2010, stroke remained the first leading cause of death and became the second leading cause of life years lost in China. Although a combination of mass and high-risk approaches for stroke prevention showed encouraging effects among the Chinese population, the stroke burden in China continues to increase over the last two decades [2, 3].

Past studies have indicated that balance and gait dysfunction is the common problems in stroke patients. This disability influences patient walking ability and quality of life. Proprioception often decreases in stroke patients, which causes patients to depend excessively on visual sense, and maybe they would wrongly rely on visual sense, which causes sensory integration disorder and abnormal compensatory strategies, leading to inappropriate body response to interference, inability to maintain stability, and decline in motor control skills. Finally, caused Falling [4, 5]. Falling is one of the most common complications suffered by stroke patients during their rehabilitation period [6]. A survey conducted by the emergency department from 1992 to 1995 in the United States found that falls are a major cause of damage. Children under the age of five fall from external causes. Accordingly, falling is more common in elderly patients over 65 years old [7], and the fifth factor which caused of death in the elderly. About two-thirds of accidental deaths, which reached 70% in elderly patients aged 75 years old and above, are caused by falls [8]. According to the survey, at least 20 million of the elderly fall 20 million times a year in China, and more than CNY 5 billion and CNY 80 to 160 billion are spent in direct medical and social costs, respectively [9, 10]. In addition, according to the national economic and social development statistical bulletin of the 2009 and 2008 statistics yearbooks, the average hospitalization cost for every cerebrovascular disease is approximately CNY 7,267.6. This value is half the annual income of urban residents and 1.5 times that of farmers[11–13]. So patients with stroke, their families, and society would shoulder heavy economic burdens because of these factors. This trend has profound financial and social consequences[14].

Hence, effectively treating stroke is important. Many studies have shown that engaging in sports is a very effective method of improving neuromuscular function. However, many movement techniques are monotonous, or certain strength exercises are beyond the patients' ability [15]. Exercise therapy, as a form of non-drug therapy, provides patients with greater benefits on physical function [16, 17]. Because brain plasticity is the basis of training exercises for patients with stroke, who are under limb function recovery. Exercise training promotes spontaneous neural functional recovery and restores brain function in patients with cerebrovascular disease to accelerate the process of functional recovery [18], which would help accelerate the rehabilitation process.domestic and foreign research has proven the benefits of exercise therapy in patients with cerebral apoplexy [16, 19]. Literature has also reported positive effects observed in patients with cerebral apoplexy after traditional Chinese exercise is used as a type of exercise therapy [8, 20–22].

Traditional Chinese exercise is a self methodology and theory of mental and physical exercise which was created by ancient Chinese people in their long life and labor. Including Taiji, Baduanjin, Yijinjing, Liuzijue and so on. As a low cost, easy to learn, highly safe, and appropriate aerobic exercise, traditional Chinese exercise is suitable for old people. In addition to these advantages, traditional Chinese exercise also offers additional benefits to the traditional method of stroke rehabilitation training by combining physical movements with mental focus and relaxation [23, 24]. Certain studies have shown that traditional Chinese exercise is a safe choice for patients with chronic illness or disabilities [25]. Tai chi as a type of representative traditional Chinese exercise, is a popular exercise method among the elderly, especially in Asia. Tai chi is divided into many genres according to different origins. These styles include the Yang, Wu, and Chen styles. Though there are differences of posture and the position of the center of gravity, all styles incorporate slowness, rhythmic movements, relaxation, mental concentration, movement coordination, and flow into the next one with elements of meditation, body awareness, and imagery while breathing deeply [26–28]. Some studies have found that practicing traditional Chinese exercise improves the ability of gait and balance and increases muscle strength for motor system function, which was useful for patients with stroke, and even for healthy adults [20–22].

Different opinions always exist. Whether traditional Chinese exercise is more beneficial in improving physical function in patients with stroke than other exercise therapy or without intervention is presently vague. A systematic review of adverse event reports in randomized trials [29] has reported certain adverse effects. These effects include muscle problems [30–32] and dizziness or hypotension [33]. Other patients feel pain in certain body parts (e.g., back, foot, and knee) or ankle sprains [23, 30, 31, 33–36]. Considering the preceding observations, traditional Chinese exercise maybe make things worse.

However, no systematic review on the efficacy of traditional Chinese exercise on the physical function of stroke patients has been found from electronic databases. Hence, this review evaluates the efficacy of traditional Chinese exercise in the physical function of stroke patients. This systematic review provides information that could help clinicians make evidence-based decisions on the use of traditional Chinese training for stroke patients.

## Methods

### Search Strategy

We did a systematic review in accordance with the PRISMA (Preferred Reporting Items for Systematic Reviews and Meta-Analyses) guidelines. For a completed PRISMA checklist, see S1 PRISMA Checklist. Randomized controlled trials (RCTs) were retrieved from the following databases: PubMed, Embase, Cochrane Library, CINAHL (EBSCO), Web of Science, and CNKI. The terms “Stroke”, “Traditional Chinese exercise”, and “randomized controlled trials” were used as search words. Full details of the search can be found in the search strategy (S1 File). This review was only limited to RCTs and had no language, year, or status restrictions. Furthermore, this review augmented the probability of finding all correlative publications discussing the influence of traditional Chinese exercise on the gait and balance of stroke patients. The protocol was registered on the international prospective register of systematic reviews(http://www.crd.york.ac.uk/PROSPERO PROSPERO, registration number: CRD42013006474).

### Inclusion criteria

The inclusion criteria were as follows:

1. **Types of studies.** This research only included randomized controlled trials(RCTs). Restrictions on language or publication date were not set.
2. **Types of participants.** The research samples included participants in accordance to the standards of stroke. Any article with participants having impaired physical functions prior to their experience with traditional Chinese exercise or who were participating in other projects at that time was included.
3. **Types of interventions.** Only trials that were compared with a treatment group practicing traditional Chinese training and a control group performing no intervention or other treatment in which either conservative treatment or a sham procedure was administered. were considered. All control treatments have specific properties that differentiate them from traditional Chinese exercise, consisted of conventional medical treatment, routine rehabilitation training. The control group was merged if three or more than three groups existed, this control group was then compared with the traditional Chinese exercise group.
4. **Types of outcome measures.** The outcome included the short physical performance battery, gait speed, step length, length difference, Functional walking scale, 2-minute step test, in addition, included the BBS, timed-up-and-go score, Fugl-Meyer, limit of stability, sensory organization tests. We categorized outcomes as short-term (≤three months), mid-term (closer to six months), and long-term (≥twelve months).

### Selection of studies

This review used the same selection criteria to screen titles by two authors (Chen BL and Li X), abstracts, and full papers of relevant articles. Articles were excluded if they did not meet the selection standard. Any dispute was resolved through discussion. A third author (Wang XQ) was subsequently asked to make final selection decisions regarding inclusion or exclusion if disagreement persisted.

### Data extraction

Data on author, year, patient characteristic, description of interventions between the control and experimental groups (e.g., sample size, interventionist strategies, and exercise frequency), outcomes, and time point were extracted from the included articles. Similarly, a third author (Wang XQ) was contacted to make the final decision if any disagreement persisted.

### Quality assessment

This study assessed the risk of bias for all included articles using the Cochrane Collaboration recommendations [37]. The following messages were evaluated: random sequence generation, allocation concealment, blinding of participants and personnel, blinding of outcome assessments, incomplete outcome data, selective reporting, and other biases. Two authors (Chen BL and Li X) were needed to complete the job and evaluate the methodological quality of all included articles. A third author (Wang XQ) was also consulted if any disagreement persisted.

In addition, we assessed the quality of evidence of outcome using the GRADE (Grades of Recommendation Assessment, Development and Evaluation) methods. The GRADE methods ranks quality of evidence into four grades: high, moderate, low and very low. And GRADE is an approach to evaluating quality of evidence and strength of recommendation from research studies based on critiquing the likelihood of bias; inconsistency of results; and indirectness of evidence.

### Statistical analysis

The Review Manager software (RevMan5.2) was used for meta-analysis. The chi-squared test and the I2 statistic were employed to evaluate heterogeneity among the studies. The fixed-effects model was utilized if the heterogeneity test indicated no significant difference (I^2^ < 50%; P > 0.1). The random-effects model was adopted otherwise. All of the continuous data obtained from the articles were included in this meta-analysis. The mean difference (MD) and 95% confidence interval (CI) were used to analyze the studies. Furthermore, P < 0.05 was considered statistically significant. Sensitivity analysis was performed to evaluate the stability of results by exclusion of each study one by one.

## Results

### Search results

**Fig 1:**
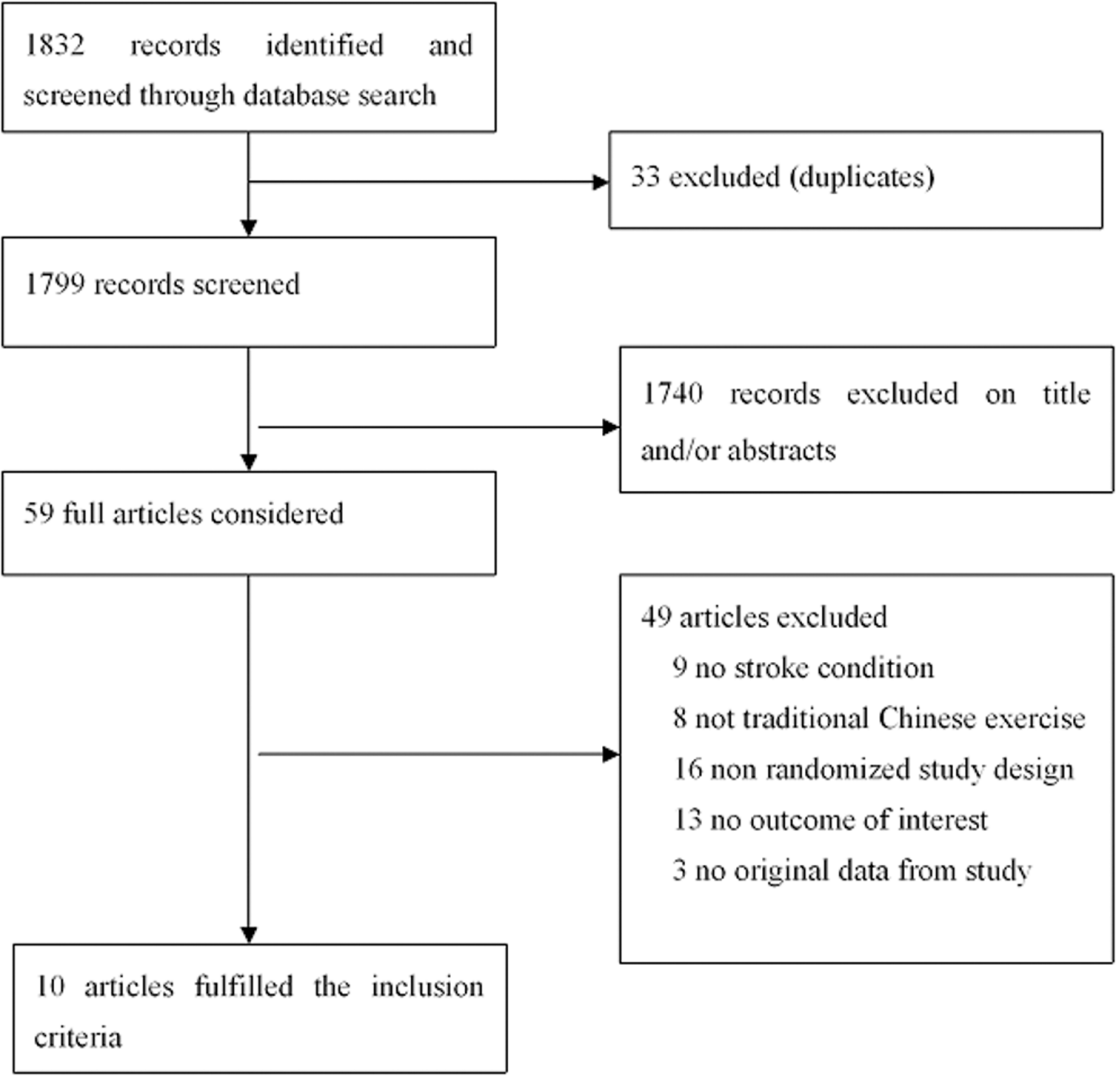
Flow chart of the study selection procedure. For details of study identification.

A total of 1833 articles were searched from PubMed, Embase, Cochrane Library, EBSCO, Web of Science, and CNKI. Accordingly, 59 potentially eligible studies were identified based on their titles and abstracts. Only 9 articles [38–46] met the inclusion criteria after the entirety of the 59 articles was reviewed. In addition, 50 articles were excluded because they either included participants without stroke or did not use traditional Chinese exercise for stroke treatment. Other studies were also excluded because the original data were no longer available from either the article or the author. Others had been excluded because they were non-randomized controlled studies or their outcome was irrelevant for the present review. Fig 1 outlines the process of identifying the qualified articles.

### Study characteristics

9 articles were included, All included articles aimed to examine the efficacy of traditional Chinese exercise on the physical function of patients with stroke. all trials were published between 2004 and 2013. And the detailed description we can see from the Table 1. All of the study were the single-center trials. Three types of interventions were used; six studies used Tai chi [38–43], two studies used Baduanjin [44, 45], and one used Yijinjing[46]. All studies were from three countries, China [38, 41–46], America [40] and Israel [39]. Data from 820 participants were extracted.

**Table 1:** Characteristics of Included Studies.

Article, Year	Patients Characteristic, Sample Size	Intervention	Duration of trial period	Outcomes	Time point
Bai Yan-jie 2011[45]	Source:60 patients diagnosed with strok(G1 = 30 G2 = 30); Mean age (SD): G1 = 53.7y(4.5); G2 = 51.3y(7.5)	G1: balancing function training+baduanjin; G2: balancing function training	two times per day for six weeks	BBS; Balance function assessment of level three	six weeks
Zhang Ming 2013[44]	Source:224 patients diagnosed with strok(G1 = 115 G2 = 106); Mean age (SD): G1 = 30~70y; G2 = 30~70y	G1: conventional medical treatment+Bobath+Baduanjin; G2: conventional medical treatment+Bobath	two times per day for six weeks	BBS	six weeks
Zhou Zugang 2013[41]	Source:68 patients diagnosed with strok(G1 = 34 G2 = 34); Mean age (SD): G1 = 62.6y(5.7); G2 = 63.3y(6.0)	G1: acupuncture+Routine rehabilitation training+Tai chi gait intensive training; G2: acupuncture+Routine rehabilitation training	six times a week for six weeks	BBS; gait parameters; FCA	six weeks
Xie Fei 2008[43]	Source:48 patients diagnosed with strok(G1 = 24 G2 = 24); Mean age (SD): G1 = 48y(3.6); G2 = 48y(3.6)	G1:TaiChi+exercise therapy; G2: exercise therapy	three times per day for two weeks	BBS	two weeks
Yang Zhibo 2013[42]	Source:100 patients diagnosed with strok(G1 = 50 G2 = 50); Mean age (SD): G1 = 54.3y(13.8); G2 = 55.2y(14.6)	G1: conventional medical treatment+TaiChi; G2: conventional medical treatment+The traditional rehabilitation training	six times a week for four weeks	BBS	four weeks
Jia Weizhong 2008[46]	Source:34 patients diagnosed with strok(G1 = 18 G2 = 16); Mean age (SD): G1 = 47.7y(15.5); G2 = 51.5y(16.37)	G1: conventional medical treatment+YiJinjing; G2: conventional medical treatment	five times a week for twelve weeks	BBS; FM-B	twelve weeks
Ruth E. Taylor-Piliae 2014[40]	Source:145 patients diagnosed with strok(G1 = 53 G2 = 44 G3 = 48); Mean age (SD): G1 = 71.5y(10.3); G2 = 69.6y(9.4);G3 = 68.2y(10.3)	G1: TaiChi; G2: (1)national fitness and; (2)Health education	one-hour class three times a week for twelve weeks	SPPB; 2-minute step test	twelve weeks
Stephanie S. Y.Au-Yeung 2009[38]	Source:136 patients diagnosed with strok(G1 = 74 G2 = 62); Mean age (SD): G1 = 61.7y(10.5); G2 = 65.9y(10.7)	G1: Tai Chi; G2: practicing general exercises	one-hour class three times a week for twelve weeks	Limit of Stability Test; the Sensory Organization test; Timed-up-and-go score	twelve weeks
Jacob Hart 2004[39]	Source:18 patients diagnosed with strok(G1 = 9 G2 = 9); Mean age (SD): G1 = 61.4y (-); G2 = 57.3 (6.8)	G1: Tai Chi; G2: group exercises focusing on improvement of balance	1 hour class twice times a week for twelve weeks	BBS; standing on one leg	six weeks; twelve weeks

**Abbreviations** G: Group; G1 = the treatment group; G2 = the control group; SD: Standard deviation; y: year; FM-B: Fugl-Meyer(FM-B); BBS: the Berg balance scale; SPPB: Short Physical Performance Battery; FCA: functional walking scale.

### Quality of the evidence

The Cochrane Collaboration recommendation indicated that every article indicated a high bias risk. A high holistic risk of bias was related to the evidence for this systematic review. Randomization was used in each article, and only two articles (22.2%) adopted the method of allocation concealment. The method of blinding the participants to the allocated treatment was used in three (22.2%) of the included articles, and six articles (66.6%) were unclear whether adopted the method of blinding of outcome assessments. All study describe the patients who withdrew from the study. The incomplete outcome in all the included articles resulted in a low bias risk. In all, all included studies were considered high risk of bias (S1 Fig). The detail of risk of bias was illustrated in Table 2.

**Table 2:** The risk of bias about included studies.

Article, Year	Random sequence generation	Allocation concealment	Blinding of Participants and personnel	Blinding of outcome assessments	Incomplete outcome data	Selective reporting	Other bias	Risk of bias
Bai Yan-jie 2011[45]	Low	high	high	unclear	Low	high	unclear	high
Zhang Ming 2013[44]	Low	high	high	unclear	Low	high	unclear	high
Zhou Zugang 2013[41]	Low	high	high	unclear	Low	unclear	unclear	high
Xie Fei 2008[43]	Low	high	high	unclear	Low	unclear	unclear	high
Yang Zhibo 2013[42]	Low	high	high	unclear	Low	unclear	unclear	high
Jia Weizhong 2008[46]	Low	high	high	unclear	low	unclear	unclear	high
Ruth E. Taylor-Piliae 2014[40]	Low	low	low	high	low	unclear	unclear	high
Stephanie S. Y.Au-Yeung 2009[38]	Low	low	low	high	low	unclear	unclear	high
Jacob Hart 2004[39]	Low	high	high	high	low	unclear	unclear	high

And all studies included were randomized controlled trial, system GRADE recommended classification method was used to evaluate the BBS, Because of the inconsistency, imprecision and Publication Bias, the level of evidence was downgraded. And there was no reason to upgrade the strength of evidence because of the limitations in design in these included studies. Finally, the result showed that the quality of the evidence (GRADE) was very low.

### Traditional Chinese exercise compared with other forms of exercise

#### Balance

In total, nine articles (820 patients) evaluated the ability of balance with the following measures: the BBS, timed-up-and-go score, Fugl-Meyer (FM-B), limit of stability, sensory organization tests, the short physical performance battery for balance.

**Fig 2:**
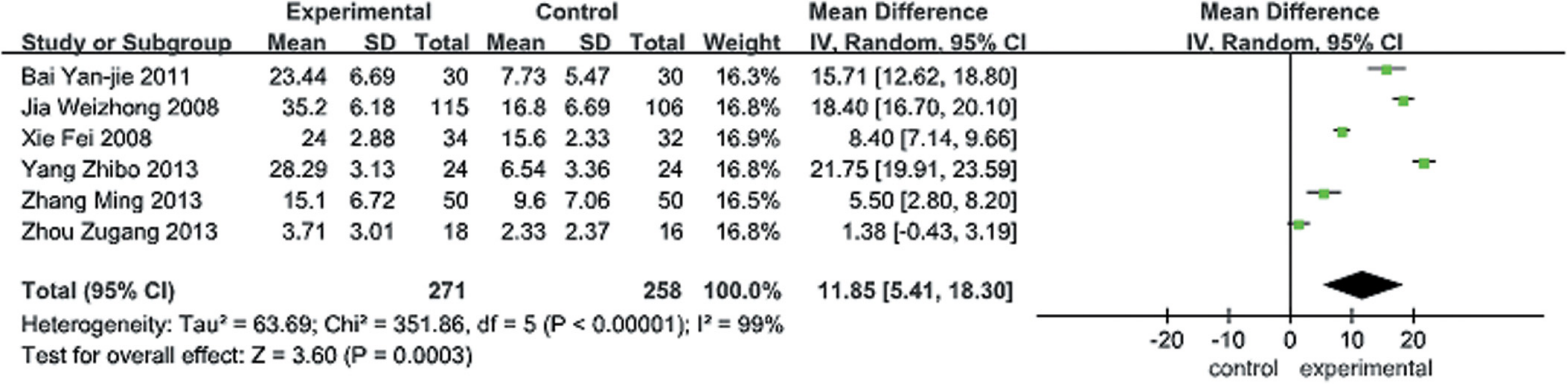
Forest plot for traditional Chinese exercise on balance in short term. Short term: not longer than 3 months; BBS = Berg balance score; SD = standard deviation; 95% CI = 95% confidence intervals; IV = inverse variance.

BBS: six trials[41–46] evaluated balance using the Berg balance scale(BBS), which was evaluated the balance by scale, and the higher score showed the better ability of balance, All of them (534 patients) were reported data at short-term follow-up period. We found significant improvements with traditional Chinese exercise for balance in the short term in a random-effects model [MD (95%CI) = 11.85 [5.41, 18.30], P < 0.00001], but the pooled estimate of effect for the BBS is extremely heterogeneous (I-squared 99%) (Fig 2). A sensitivity analysis was performed and there was no substantial modification of our estimates after exclusion of individual study one by one.

Other outcomes: In addition to BBS, we found that one article (34 patients) used the the FM-B (which was similar with BBS) to demonstrate that Yijinjing improved the ability of balance for stroke patients (P < 0.05) [46]. And the limit of stability, sensory organization tests and the timed-up-and-go score to evaluate the standing balance of stroke patients (136 patients), all data indicated that tai chi was more effective than general exercises (P < 0.05) [38]. Another article(145 patients) showed that the significantly improved between the effects of traditional Chinese and general exercises were observed using the the short physical performance battery (P < 0.05) [40].

However, there is a article showed that some other form (physiotherapy exercises focused on balance) significant improved balance ability for stroke patients compared with tai chi chuan practice (Berg test = 0.01, Romberg, and standing on one leg) [39].

#### Gait

We used more than one measure of gait variables, and we preferentially included the following measures: the short physical performance battery for gait, gait speed, step length, length difference, Functional walking scale (FCA), 2-minute step test. And with the balance different, only individual gait variables was found, so we can't make the forest plot to quantification. We found that a article assessed the gait variables using the short physical performance battery and 2-minute step test (P < 0.05), significant differences were observed between groups at short-term (twelve weeks) [39]. In addition, another article showed that after 3 weeks and 6 weeks' treatment, the gait parameters(except the step length) and FCA were significantly improved and the difference had statistical significance (p<0.05) between the groups, and the Tai Chi group didn't have obvious improvement in step length within 3 weeks, only after 6 weeks did it show the obvious improvement. Significantly (P < 0.05) [41].

## Discussion

**Fig 3:**
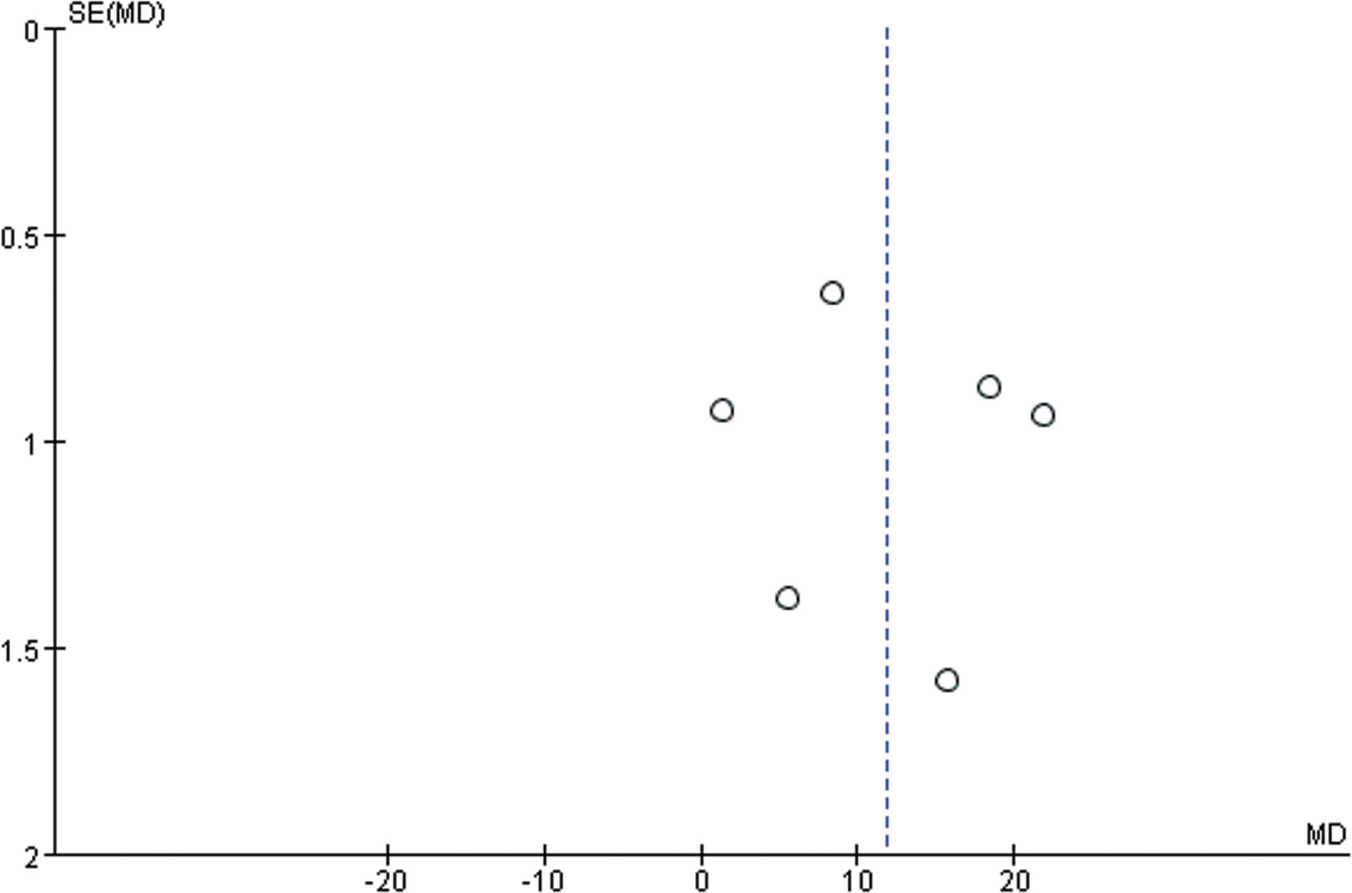
Funnel plot for traditional Chinese exercise on balance in short term. Short term: not longer than 3 months.

Previous reviews have examined the results of traditional Chinese exercise versus some other forms of exercise or non-intervention on cardiorespiratory fitness or upper-extremity dysfunction with stroke patients or health adults [20, 23]. But none has focused on gait and balance for stroke patients. Gait and balance are important for stroke patients to prevent falls and worse consequences. Therefore, this review collated the evidence from a large number of trials, which evaluated the effect of traditional Chinese exercise on gait and balance for stroke. This meta-analysis includes 820 patients, who participated in 9 RCTs. And all of the included articles belong to short-term (≤ 3 months). Five even less than three months [41–45]. no adverse events are reported in the 9 RCTs included in the meta-analysis. And according to the system GREADE recommended, the quality of the evidence (GRADE) of BBS was very low, and the reason maybe extremely heterogeneous (I-squared 99%), imprecision or incomplete data, and publication bias according to the Funnel plot (Fig 3), because the dots represent the mean differences of individual trial estimates and the dotted line the point estimate of the mean effect indicating benefit from traditional Chinese exercise. The distribution of these dots to the right of the dotted line suggests that there may be the equivalent number of ‘negative’ trials that have not been included in this analysis [47].

The results of this meta-analysis show that traditional Chinese exercise is better than other forms of exercise in improving the balance of patients with stroke significantly at short-term. Because of the disunity outcome indicators, we couldn’t finish the forest plot about gait. But, according to the result, Three trials [39, 40] showed a tendency to improve in gait [39–41]. So an increasing number of studies have shown that traditional Chinese exercise is effective for improving the ability of balance for patients with stroke, and maybe the gait. Some researchers suggest that traditional Chinese exercise improves joint stability, and the improved limits of stability are associated with improved functional performance [48]. In addition, no adverse events were reported between groups in this meta-analysis. But certain articles [29] assert that tai chi cannot cause terrible adverse events although it may be connected with minor musculoskeletal aches and pains. Attention should be paid to the appropriate method and strength to use when practicing this traditional Chinese exercise. However, poor and inconsistent reporting of adverse events greatly limits the conclusions determined as regards the safety of practicing tai chi.

The pooled estimate of effect for the BBS (Fig 2) is extremely heterogeneous (I-squared 99%). Maybe there are probably important clinical and methodological differences among studies that influence the difference between intervention and control(s).

1. Different in populations: In the six articles, some difference existed in inclusion criteria. Especially about the duration of stroke, the patients with convalescence (≤6 months) were included [41–45], and another three articles were the patients with period of stroke sequel [48]. Maybe the different baseline caused the extremely heterogeneous.
2. Different types: Traditional Chinese exercise does not only include tai chi but also other forms, such as baduanjin, qigong, wuqinxi. liuzijue, and so on. Even tai chi also has a lot of branches. In the six articles which was included, three articles’ intervention methods are Tai chi (one of them is Yang-style Tai chi, but another two were unclear), two articles are Baduanjin, and the rest is Yijinjing. Different forms of traditional Chinese exercise focus on different emphasis. This maybe a factor to cause the heterogeneity.
3. Different in experimental and control group: there are some difference in grouping for theses article. conventional medical treatment combined with traditional exercises exercise as a treatment for three articles [42, 44, 46], The remaining three articles [41, 43, 45], respectively traditional Chinese exercise combined with balancing function training, acupuncture and exercise therapy. Different treatments may lead to such the result.
4. Different in intensity and duration: Different intensity and duration may lead to different results. Accordingly, we find that different frequency of training was used for included articles. Bai Yan-jie and Zhang Ming [44, 45] took two times per day for six weeks, Zhou Zugang [41] took six times a week for six weeks, Xie Fei [43] choosed three times per day for two weeks, the remaining two are respectively six times a week for four weeks and five times a week for twelve weeks (Table 1).

### Strengths

Our study has several strengths. First, a detailed search strategy is employed to collect data by searching different electronic citation databases and trial registries. Moreover, no language limitation or publication date is set to ensure inclusion of as much data as possible from appropriate studies. Two reviewers independently chose, extracted, and evaluated the quality of data to reduce bias and transcription errors. and this work is the first systematic review and meta-analysis to evaluate the efficacy of traditional Chinese exercise on gait and balance compared with other forms of exercise, it could provide a evidence for clinical workers that would help them make better clinical decisions.

### Limitations

However, this systematic review also has certain limitations. First, no high-quality research report is included in this systematic review. According to the Cochrane Collaboration recommendations, nine articles in the systematic review have low qualities with a high bias risk. Second, because of the poor and non-uniform outcome. there is no distinction between primary and secondary outcomes, so maybe the results are at higher risk of chance findings due to multiplicity of testing and selective reporting. Third, too many inconsonant indices are used to make the forest plot, especially for gait variables. Accordingly, only the BBS are utilized in the forest plot. Moreover, other indices, such as functional walking scale, balance subscale of the FM-B, and timed-up-and-go score, are not suitable for the complete forest plot. Hence, the declarative description is selected. The probability of publication bias is the fourth limitation. We have tried to reduce the probability of publication bias through a substantial database search. However, we did not search for unpublished articles. Therefore, we may have missed some important data.

### Implications for research

Compared to other forms of exercise, traditional Chinese exercise may be more effective in improving the gait and balance of patients with stroke in short term, which were the only factors observed in this review. Inadition to, attention should also be given to other points concerning traditional Chinese exercise for patients with stroke, these aspects include fall rates, quality of life, and cognition. However, these consequences are obtained by certain examples of low-quality data, and more explicit articles are needed to confirm these consequences.

## Conclusions

Based on our results, we make the following recommendations. The positive findings of this study suggest traditional Chinese exercise has beneficial effects on the balance ability in short term, and maybe a tendency to improve gait. However, the conclusion was drawn according to the extreme heterogeneity, and evidence of better quality and from a larger sample size is required. In the future, uniform and accepted outcome of gait should be used in traditional Chinese exercise for patients with stroke. Systematic data collection at short, mid-, and long-term follow-up is essential.

## Supporting Information

S1 PRISMA Checklist PRISMA Checklist of this meta-analysis. (DOC)

S1 Fig Risk of bias summary using the Cochrane Risk of Bias tool. (TIF)

S1 File Search strategies for all databases. (DOCX)
