# Supplementary figures and images for: Effect of Traditional Chinese Exercise on Gait and Balance for Stroke: A Systematic Review and Meta-Analysis
Source: PLoS One. 2015 Aug 20;10(8):e0135932. doi: 10.1371/journal.pone.0135932 (PMC4546302; doi:10.1371/journal.pone.0135932)

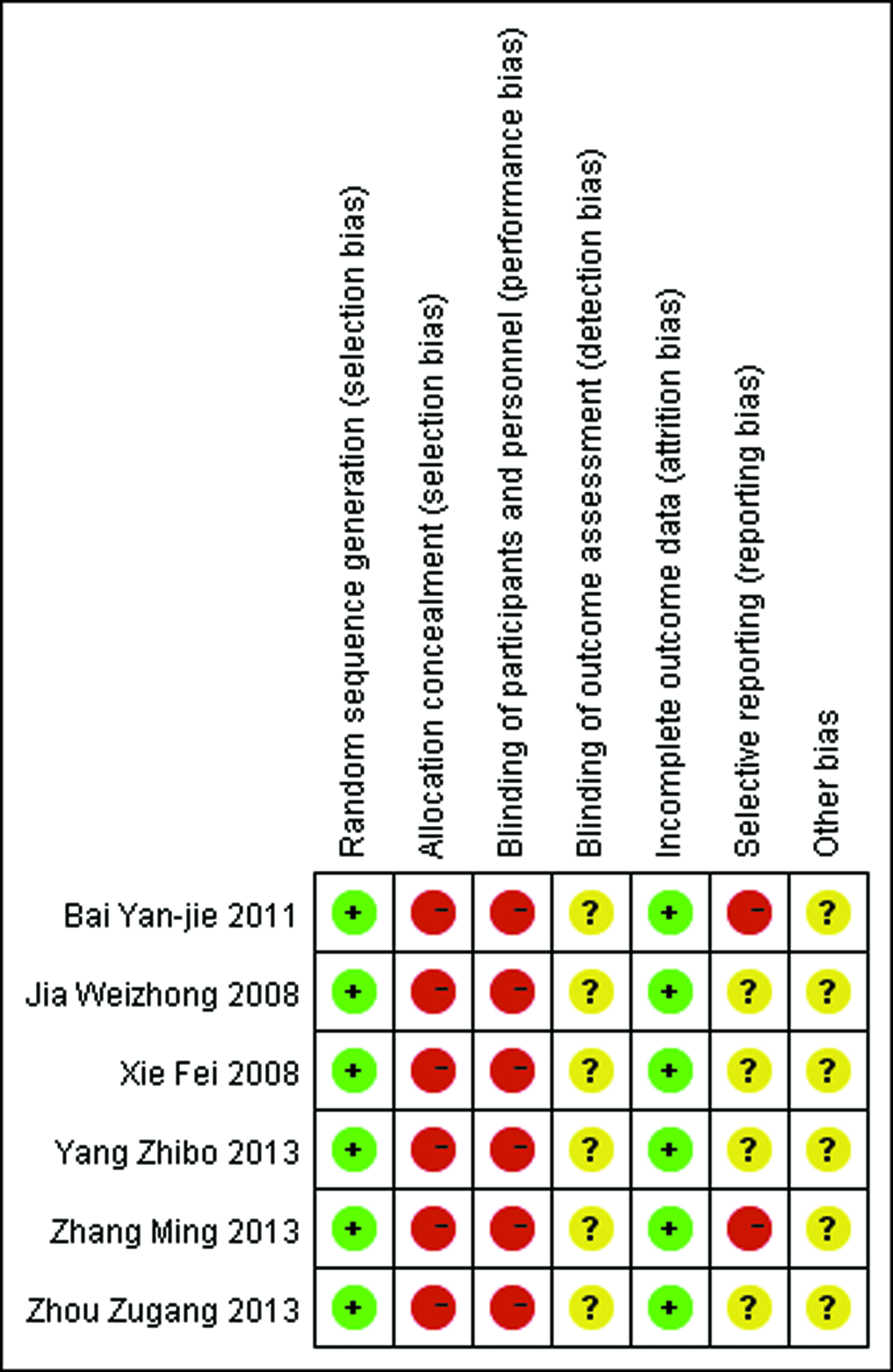

Supplement: S1 Fig — (TIF) [file pone.0135932.s002.tif]
