# Supplementary material for: Effect of Traditional Chinese Exercise on Gait and Balance for Stroke: A Systematic Review and Meta-Analysis
Source: PLoS One. 2015 Aug 20;10(8):e0135932. doi: 10.1371/journal.pone.0135932 (PMC4546302; doi:10.1371/journal.pone.0135932)
Supplement: S1 File — (DOCX) [file pone.0135932.s003.docx]

Effect of Traditional Chinese exercise on gait and balance for Stroke: systematic review and meta-analysis

**Search strategies for all databases**

1. Search Strategy for Pubmed:

#1 "Single-Blind Method"[Mesh] OR "Double-Blind Method"[Mesh] OR "Randomized Controlled Trials as Topic"[Mesh] OR "Randomized Controlled Trial" [Publication Type] OR "Intention to Treat Analysis"[Mesh] OR "Controlled Clinical Trials as Topic"[Mesh] OR "Clinical Trials as Topic"[Mesh] OR "Clinical Trial" [Publication Type]

#2 "random*"[Text Word] OR allocation[Text Word] OR "random allocation"[Text Word] OR placebo[Text Word] OR single blind[Text Word] OR double blind[Text Word] OR "randomized controlled trial*"[Text Word] OR RCT[Text Word]

#3 randomized controlled trial[Publication Type]

#4 #1 OR #2 OR #3

#5 animals NOT humans

#6 #4 NOT #5

#7 Stroke[Text Word] OR "cerebrovascular accident "[Text Word] OR CVA[Text Word] OR “cerebral apoplexy” [Text Word] OR " cerebral arterial thrombosis "[Text Word]

#8 “ischemia stroke” [Text Word] OR "cerebral ischemic stroke "[Text Word] OR "cerebral thrombosis "[Text Word] OR “cerebral infaction” [Text Word] OR “Cerebral thrombus” [Text Word] OR “cerebral embolism” [Text Word] OR “cerebral infarction” [Text Word] OR “cerebral thrombosis” [Text Word]

#9 "hemorrhagic stroke"[Text Word] OR hematencephalon[Text Word] OR "cerebral hemorrhage"[Text Word] OR “intracerebral hemorrhage” [Text Word] OR ICH[Text Word] OR "brain hemorrhage"[Text Word] OR encephalorrhagia[Text Word] OR "Subarachnoid hemorrhage"[Text Word] OR "SAH"[Text Word] OR “Subarachnoidal bleeding” [Text Word] OR “Traumatic subarachnoid haemorrhage” [Text Word]

#10 #7 OR #8 OR #9

#11  tai chi[Title/Abstract] OR "taiji*"[Title/Abstract] OR qigong[Title/Abstract] OR wuqinxi[Title/Abstract] OR baduanjin[Title/Abstract] OR "traditional exercise"[Title/Abstract] OR traditional chinese medicine[Title/Abstract] OR "chinese traditional exercise" OR "traditional chinese exercise" OR "chinese exercise"

#12 #6 AND #10 AND #11

2. Search Strategy for EMBASE:

#1 'randomization'/exp OR 'placebo'/exp OR 'placebo effect'/exp OR 'single blind procedure'/exp OR 'double blind procedure'/exp OR 'randomized controlled trial'/exp OR 'randomized controlled trial (topic)'/exp OR 'controlled clinical trial'/exp OR 'controlled clinical trial (topic)'/exp OR 'clinical trial'/exp OR 'clinical trial (topic)'/exp

#2 random*:ab,ti OR allocation:ab,ti OR "random allocation":ab,ti OR placebo:ab,ti OR single blind:ab,ti OR double blind:ab,ti OR randomised controlled trial*:ab,ti OR randomized controlled trial*:ab,ti OR RCT:ab,ti OR clinical trial*:ab,ti

#3 #1 OR #2

#4 Stroke OR " cerebrovascular accident " OR CVA OR “cerebral apoplexy” OR " cerebral arterial thrombosis " OR “ischemia stroke” OR "cerebral ischemic stroke " OR "cerebral thrombosis " OR “cerebral infaction” OR “Cerebral thrombus” OR " trombosis celebral " OR “cerebral embolism” OR “cerebral infarction” OR "cerebrl bolism " OR “cerebral thrombosis” OR "hemorrhagic stroke" OR "hemorrhage apoplexy" OR “hemorrhagic cerebral apoplexy” OR hematencephalon OR "cerebral hemorrhage" OR “intracerebral hemorrhage” OR ICH OR "brain hemorrhage " OR encephalorrhagia OR "Subarachnoid hemorrhage" OR "SAH" OR “Subarachnoidal bleeding” OR “Traumatic subarachnoid haemorrhage”

#5 tai chi:ab,ti OR taiji*:ab,ti OR qigong:ab,ti OR liuzijue:ab,ti OR wuqinxi:ab,ti OR yijinjing:ab,ti OR baduanjin:ab,ti OR traditional exercise:ab,ti OR chinese traditional exercise:ab,ti OR traditional chinese exercise:ab,ti OR chinese exercise:ab,ti

#6 #3 AND #4 AND #5

3. Search Strategy for Cochrane Library

#1 "random*" or allocation or "random allocation" or placebo or single blind or double blind or "randomized controlled trial*" or RCT or "clinical trial*"

#2 randomized controlled trial:pt or clinical trial:pt

#3 Stroke:ti,ab,kw OR "cerebrovascular accident ":ti,ab,kw OR CVA OR “cerebral apoplexy” :ti,ab,kw OR " cerebral arterial thrombosis ":ti,ab,kw OR “ischemia stroke” :ti,ab,kw OR "cerebral ischemic stroke ":ti,ab,kw OR "cerebral thrombosis ":ti,ab,kw OR “cerebral infaction” :ti,ab,kw OR “Cerebral thrombus” :ti,ab,kw OR “cerebral embolism” :ti,ab,kw OR “cerebral infarction” :ti,ab,kw OR “cerebral thrombosis” :ti,ab,kw OR "hemorrhagic stroke":ti,ab,kw OR hematencephalon:ti,ab,kw OR "cerebral hemorrhage":ti,ab,kw OR “intracerebral hemorrhage” :ti,ab,kw OR ICH:ti,ab,kw OR "brain hemorrhage":ti,ab,kw OR encephalorrhagia:ti,ab,kw OR "Subarachnoid hemorrhage":ti,ab,kw OR "SAH":ti,ab,kw OR “Subarachnoidal bleeding” :ti,ab,kw OR “Traumatic subarachnoid haemorrhage” :ti,ab,kw

#4 tai chi:ti,ab,kw OR taiji*:ti,ab,kw OR qigong:ti,ab,kw OR liuzijue:ti,ab,kw OR wuqinxi:ti,ab,kw OR yijinjing:ti,ab,kw OR baduanjin:ti,ab,kw OR traditional exercise:ti,ab,kw OR chinese traditional exercise:ti,ab,kw OR traditional chinese exercise:ti,ab,kw OR chinese exercise:ti,ab,kw

#5 #1 and #2 and #3 and #4

4. Search Strategy for CINAHL (Ebsco)

S1 MH("Random Assignment" OR "Placebos" OR "Placebo Effect" OR "Single-Blind Studies" OR "Double-Blind Studies" OR "Triple-Blind Studies" OR "Randomized Controlled Trials" OR "comparative studies" OR "Evaluation Research" OR "Prospective Studies" OR "crossover Design" OR "Prospective Studies" OR "Clinical Trials" OR "Clinical Trial Registry")

S2 TX(random$ OR allocation OR "random allocation" OR placebo$ OR single blind OR double blind OR "randomi?ed controlled trial*" OR "controlled clinical trial*" OR "comparative study" OR "evaluation stud*" OR "follow-up stud*" OR "prospective stud*" OR "cross-over stud*" OR control$ OR prospectiv$ OR volunteer$ OR "RCT" OR "clinical trial*")

S3 PT( randomized controlled trial OR "clinical trial*")

S4 S1 OR S2 OR S3

S5 TX(Stroke OR "cerebrovascular accident " OR CVA OR “cerebral apoplexy” OR " cerebral arterial thrombosis " OR “ischemia stroke” OR "cerebral ischemic stroke " OR "cerebral thrombosis " OR “cerebral infaction” OR “Cerebral thrombus” OR “cerebral embolism” OR “cerebral infarction” OR “cerebral thrombosis” OR "hemorrhagic stroke" OR hematencephalon OR "cerebral hemorrhage" OR “intracerebral hemorrhage” OR ICH OR "brain hemorrhage" OR encephalorrhagia OR "Subarachnoid hemorrhage" OR "SAH" OR “Subarachnoidal bleeding” OR “Traumatic subarachnoid haemorrhage”)

S6 AB(tai chi OR taiji OR qigong OR liuzijue OR wuqinxi OR yijinjing OR baduanjin OR traditional exercise OR chinese traditional exercise OR traditional chinese exercise OR chinese exercise)

S7 S4 AND S5 AND S6

5. Search Strategy for web of science

#1 TS=("random*" OR allocation OR "random allocation" OR placebo OR single blind OR single blind method OR double blind OR double blind method OR "randomized controlled trial*" OR "randomised controlled trial*" OR "RCT" OR "clinical trial*")

#2 TS=(tai chi OR taiji OR qigong OR liuzijue OR wuqinxi OR yijinjing OR baduanjin OR traditional exercise OR chinese traditional exercise OR traditional Chinese exercise OR Chinese exercise)

#3 TS=( Stroke OR " cerebrovascular accident " OR CVA OR “cerebral apoplexy” OR " cerebral arterial thrombosis " OR “ischemia stroke” OR "cerebral ischemic stroke " OR "cerebral thrombosis " OR “cerebral infaction” OR “Cerebral thrombus” OR " trombosis celebral " OR “cerebral embolism” OR “cerebral infarction” OR "cerebral bolism " OR “cerebral thrombosis” OR "hemorrhagic stroke" OR "hemorrhage apoplexy" OR “hemorrhagic cerebral apoplexy” OR hematencephalon OR "cerebral hemorrhage" OR “intracerebral hemorrhage” OR ICH OR "brain hemorrhage " OR encephalorrhagia OR "Subarachnoid hemorrhage" OR "SAH" OR “Subarachnoidal bleeding” OR “Traumatic subarachnoid haemorrhage”)

### #4 #1 AND #2 AND #3

)

Timespan=All years. Databases=SCI-EXPANDED, SSCI, A&HCI, CPCI-S, CPCI-SSH.

1. Search Strategy for CNKI

#1 SU=随机 OR SU=随机分配 OR SU=随机对照 OR SU=对照 OR SU=盲法 OR SU=单盲 OR SU=双盲 OR SU=随机对照试验 OR SU=随机对照研究 OR SU=临床试验 OR SU=临床观察 OR SU=临床研究（精确匹配）

#2 SU=脑卒中OR SU=缺血性脑卒中OR SU=脑血栓OR SU=脑栓塞OR SU=出血性脑卒中OR SU=脑出血OR SU=蛛网膜下腔出血（精确匹配）

#3 SU=太极拳 OR SU=气功 OR SU=六字诀 OR SU=易筋经 OR SU=五禽戏 OR SU=八段锦 OR SU=传统训练 OR SU=中国传统运动训练 OR SU=中国训练（精确匹配）

#4 #1 AND #2 AND #3
